# An Improved Split-Ring Resonator-Based Sensor for Microfluidic Applications

**DOI:** 10.3390/s22218534

**Published:** 2022-11-05

**Authors:** Wei Ye, Da-Wei Wang, Jing Wang, Gaofeng Wang, Wen-Sheng Zhao

**Affiliations:** MOE Engineering Research Center of Smart Micro-Sensors and Micro-Systems, School of Electronics and Information, Hangzhou Dianzi University, Hangzhou 310018, China

**Keywords:** split-ring resonator, defected ground structure, interdigital capacitor, microfluidic channel, sensor

## Abstract

This study proposes an ultrahigh-sensitivity split-ring resonator-based microwave sensor for retrieving the complex permittivity of liquid samples. An interdigital capacitor structure was used to expand the sensing area and the sensitivity. A defected ground structure and A parallel dual split-ring resonator were introduced to improve the quality factor. A polydimethylsiloxane microfluidic channel substrate was placed above the interdigital capacitor structure. The channel route coincided with the interdigital gap to fully utilize the strong electric field. Ethanol–water solutions with varying ethanol fractions were injected into the channel as the testing liquid. It was demonstrated that the variation in resonant frequency can be used to retrieve the dielectric properties of liquid samples. The proposed sensor used a small liquid volume of ~0.68 μL and provided values in good agreement with the reference data.

## 1. Introduction

Microwave dielectric spectroscopy has advantages in detecting and identifying materials due to its real-time measurements, non-invasiveness, high sensitivity, and robustness [1]. Planar resonator-based microwave sensors have been applied in many testing and characterization scenarios, including displacement and rotation sensing [2], crack detection of metal and non-metal materials [3], medical settings [4], and dielectric constant measurements for solid dielectrics and liquid chemicals [5,6,7,8].

The working principle of resonator-based sensor is as follows. When a sample is placed in the sensing area, it will induce shifts of resonant frequency and notch magnitude, which could be used to retrieve the dielectric properties of the loaded sample. The mainstream resonators include split-ring resonators (SRR), complementary SRRs (CSRR) [9,10], electric-LC (ELC) resonators [11], defected ground structures (DGS) [12], stepped impedance resonators [13], and spiral resonators [14]. Specific functionalities such as dual mode, compact size, and differential measurements require further investigation.

In the past several years, considerable research efforts have been put into developing microwave sensors for detecting mixed solutions. SRR and CSRR are the most widely used resonators in the design of microwave planar sensors [15,16]. In the implementation of CSRR-based sensors, a microfluidic channel chip should be placed on the ground plane to allow liquid injection. However, the applications of planar microwave sensors usually require an additional periphery readout circuitry [17,18]. The assembly of the microfluidic channel chip on the ground plane would make the CSRR-based sensors difficult to integrate with the periphery circuitry.

In comparison with the CSRR structure, SRRs are easy to integrate but face the limitations of a small sensing area and a low quality factor. To improve the sensing area, new types of SRR-based sensors were developed. In [19], an SRR-based sensor was realized by incorporating an SRR within a square loop; however, it required a large amount of liquid due to its intrusive detection method. By loading two identical SRRs into a microstrip splitter/combiner configuration, a differential-mode sensor was designed in [20]. Further, a triple SRRs structure was proposed in [21], with the substrate punched in the gap of the outermost SRR to allow the liquid sample to flow vertically. An inter-digital structure was incorporated into an SRR to increase the sensing area [22]. On the other hand, several efforts have been devoted to improving the sensor quality factor. A three-stage coupled SRR structure was employed in [23], and an active feedback loop was introduced in [9] to increase the sensor resolution. However, the sensitivities of these sensors were relatively low due to their limited sensing area.

In this study, we propose an electrically small microwave planar sensor by combining an SRR and an interdigital capacitor (IDC). By etching a rectangular area on the ground plane below the IDC-SRR structure and utilizing four metallic vias, a dual-SRR structure was realized, and both the sensor sensitivity and the quality factor were improved. The rest of this paper is organized as follows. In Section 2, circuit models and optimization of the SRR-based sensor are presented. Then, a polydimethylsiloxane (PDMS) microfluidic channel chip was attached on the proposed dual-SRR, and the simulated results are described. In Section 3, a prototype of the proposed sensor was fabricated and tested for experimental verification. Some conclusions are finally drawn in Section 4.

## 2. Working Principle and Sensor Design

In this section, the working principle and optimization of an SRR-based planar microwave sensor are introduced and discussed. A complete comparison of all proposed designs is made at the end of this section.

### 2.1. IDC-SRR-Based Sensor

**Figure 1:**
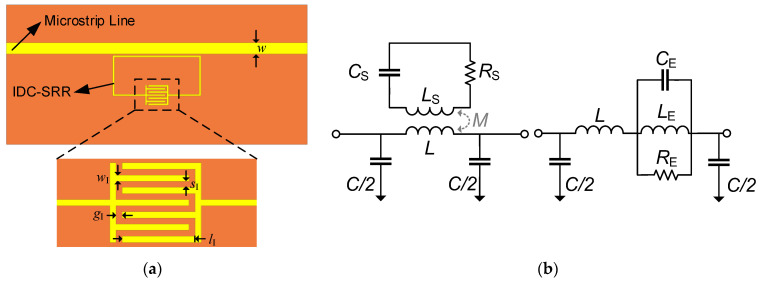
(**a**) Schematic and (**b**) equivalent circuit model of the IDC-SRR-based sensor. The geometrical dimensions are *l*_I_ = 2.4 mm and *w*_I_ = *s*_I_ = *g*_I_ = 0.2 mm.

Compared with the CSRR-based sensor, whose sensitivity is also influenced by the coupling capacitance between the CSRR and the microstrip line [16], the resonant frequency of an SRR-based sensor only depends on the effective capacitance of the SRR [24]. Therefore, it is intuitive focus on the design of the SRR effective capacitance. Here, the IDC structure was employed in the SRR design to increase the sensing area and sensitivity, as shown in Figure 1a. The equivalent circuit model is shown in Figure 1b. In general, the microstrip line can be modeled by the inductance L and the capacitance C, as shown in Figure 1b. At the resonant frequency, the IDC-SRR can be represented by a series combination of inductance $L_{S}$, capacitance $C_{S}$ and resistance $R_{S}$ of the SRR [25]. M denotes the mutual inductance between the microstrip line and the SRR. As shown in Figure 1b, the circuit model was simplified as a parallel model with $L_{E}=\omega^{2}M^{2}C_{S}$, $C_{E}=L_{S}/\left(\omega^{2}M^{2}\right)$ and $R_{E}=\omega^{2}M^{2}/C_{S}$, where ω is the angular frequency [24]. The equivalent impedance $Z_{S}$ can be calculated by $Z_{S}={\left(1/R_{E}+1/\left(j\omega L_{E}\right)+j\omega C_{E}\right)}^{-1}$ [26]. For a two-port network, the transmission coefficient $S_{21}$ can be expressed as $S_{21}=Z_{C}∥Z_{0}/Z_{C}∥Z_{0}+Z_{S}+Z_{L}$, where $Z_{C}=2/\left(j\omega C\right),$ and $Z_{L}=j\omega L$. $Z_{C}$ and $Z_{L}$ are the impedances generated by parallel capacitance and line inductance, respectively. Here, $R_{S}$ is deliberately ignored for convenience, and the resonant frequency can be defined as

(1) $$f_{0}=\frac{1}{2\pi \sqrt{L_{E}C_{E}}}=\frac{1}{2\pi \sqrt{L_{S}C_{S}}}$$

**Figure 2:**
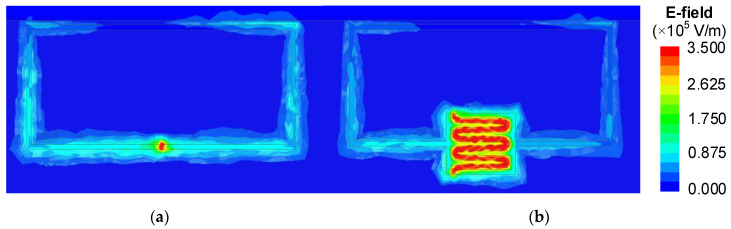
Magnitude distributions of the electric field on the top microstrip coupled with (**a**) a traditional SRR and (**b**) the IDC-SRR at the resonant frequency.

The IDC capacitance $C_{I}$ can be regarded as several pairs of parallel plate capacitors, with a uniform strong electric field between the plates. It is easy to implement a microfluidic channel along the meander gap. As shown in Figure 2, although the electric field is confined at the gap of a traditional SRR, it is difficult to load a liquid sample into such small sensing area. By introducing an IDC structure in the SRR, the sensing area can be increased to allow the loading of a liquid.

### 2.2. DGS-IDC-SRR-Based Sensor

**Figure 3:**
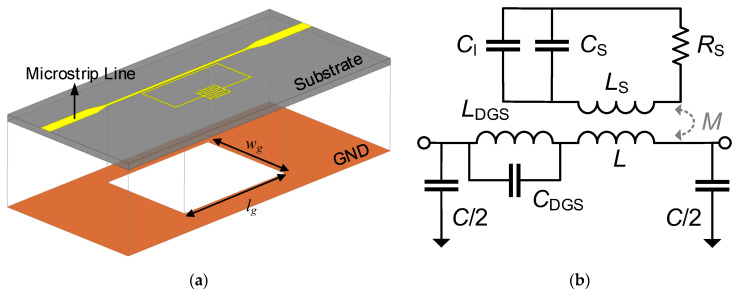
(**a**) Schematic and (**b**) equivalent circuit model of the DGS-IDC-SRR-based sensor. The geometrical dimensions are *w*_g_ = 11.5 mm and *l*_g_ = 15 mm.

It is worth noting that the applications of SRR-based sensors are usually limited by their low quality factor. To improve the sensor resolution, a DGS was introduced, and the microstrip line width was reduced in the sensor design to increase the notch magnitude, as shown in Figure 3a. In the figure, $w_{g}$ and $l_{g}$ are the width and the length of the DGS. The reduced microstrip line width would increase the impedance, thereby increasing the coupling between the SRR and the microstrip line [26]. Moreover, the etched structure on the ground plane would introduce an effective inductance $L_{\mathrm{DGS}}$ and an effective capacitance $C_{\mathrm{DGS}}$ into the model [27], as shown in Figure 3b. The transmission coefficient of the DGS-IDC-SRR-based sensor can be expressed as $S_{21}={\left[1+\left(Z_{L}+Z_{\mathrm{DGS}}+Z_{S}\left(\varepsilon_{r}\right)\right)\left(1/Z_{C}+1/Z_{0}\right)\right]}^{-1}$, where $Z_{\mathrm{DGS}}$ represents the impedance induced by the DGS. It is evident that the total impedance increased, and therefore, the notch magnitude could be improved.

### 2.3. DGS-IDC-DSRR-Based Sensor

**Figure 4:**
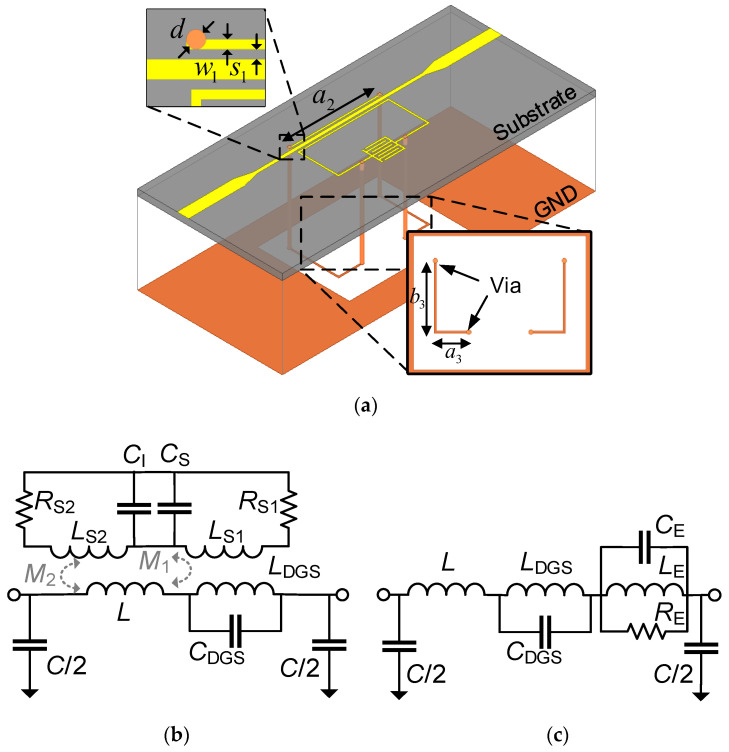
(**a**) Schematic of the complete sensor and (**b**) its equivalent circuit model with the DGS structure. (**c**) Simplified model of the whole structure. The geometrical dimensions are *d* = 0.3 mm, *a*_2_ = 11.6 mm, *a*_3_ = 3.2 mm, and *b*_3_ = 6.4 mm.

On the basis of the DGS-IDC-SRR structure, an additional ring was added in the resonator through four metallic vias, as shown in Figure 4a. In this way, a dual-SRR (DSRR) structure was obtained. Figure 4b,c show the equivalent circuit model and the corresponding simplified model of the proposed DGS-IDC-DSRR-based sensor. It is evident that the added ring would cause a mutual inductance, which increased the SRR impedance $Z_{S}$ and, ultimately, the notch depth.

**Figure 5:**
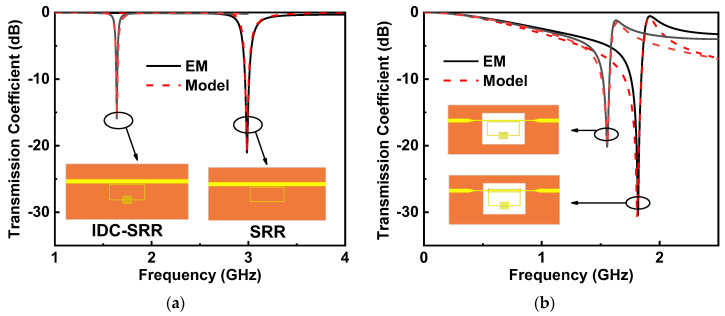
Comparison of the circuit and EM simulation results for the bare sensor. (**a**) SRR, IDC-SRR, (**b**) DGS-IDC-SRR, and DGS-IDC-DSRR.

In this study, the 0.762 mm thick dielectric substrate Rogers RO4350B with a dielectric constant of 3.66 and a loss tangent of 0.0031 was selected. The substrate dimension was set as 39 mm × 18 mm. The full-wave electromagnetic simulator ANSYS HFSS was used to obtain the transmission coefficient. As shown in Figure 5, the simulated results obtained by HFSS and circuit model agreed well with each other.

**Figure 6:**
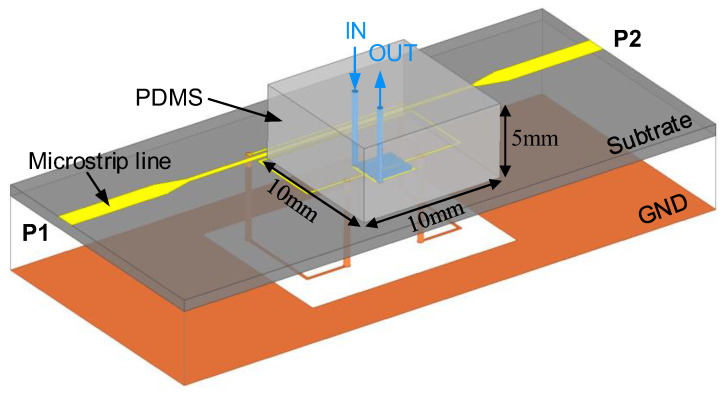
Schematic of the DGS-IDC-DSRR-based sensor for microfluidic applications.

Further, to allow liquid flowing, a PDMS microfluidic channel chip with external size of 10 mm × 10 mm × 5 mm was attached to the proposed sensor, as shown in Figure 6. The microfluidic channel route coincided with the meander gap of the IDC, and the channel height was 0.2 mm. There were two cylindrical holes with a diameter of 0.3 mm at both ends of the channel. For comparative analysis, a liquid sample with varying permittivity was injected into the channel. The relative frequency shift is defined as

(2) $$f_{r}=\frac{f_{o}-f_{\mathrm{loaded}}}{f_{o}}=\frac{\frac{1}{\sqrt{2\pi \left(L_{E}C_{0}\right)}}-\frac{1}{\sqrt{2\pi \left(L_{E}C_{\mathrm{loaded}}\right)}}}{\frac{1}{\sqrt{2\pi \left(L_{E}C_{0}\right)}}}=1-\sqrt{\frac{1}{1+\frac{C_{2}}{C_{1}}\cdot \frac{\varepsilon_{\mathrm{LUT}}-\varepsilon_{\mathrm{Air}}}{\varepsilon_{r}+\varepsilon_{\mathrm{Air}}}}}$$

where $f_{o}$ and $f_{\mathrm{loaded}}$ are the resonant frequencies of the unloaded sensor and of the sensor loaded with the liquid sample, respectively. Here, $C_{1}$ is the capacitive effect induced by air on one side of the sensing area, and $C_{2}$ stands for the capacitive effect of air in the microfluidic channel. It is worth noting that, different from the microfluidic channel of traditional SRR-based sensors, the liquid sample loaded in this customized microfluidic channel covered the gap of the IDC, so that all the LUT could achieve a high utilization rate due to their uniform distribution in the region of a strong E-field.

**Figure 7:**
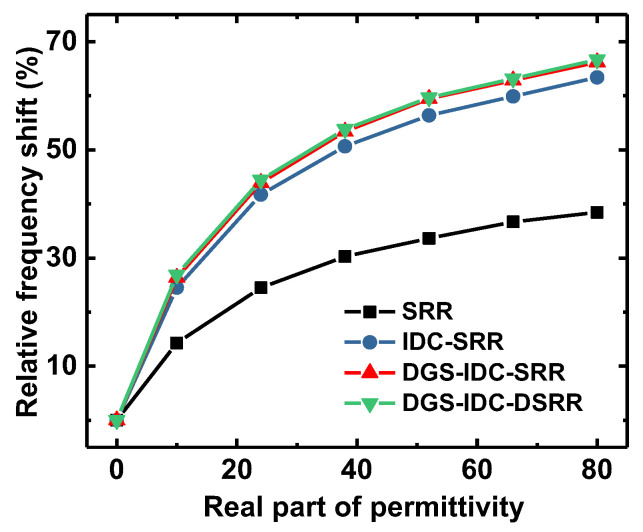
Simulated relative frequency shift for four sensors.

Figure 7 shows the relative frequency shift of four sensors with respect to the loaded liquid sample. The permittivity of the loaded liquid sample increased from 1 (air) to 81 (distilled water). It is evident that the utilization of an IDC in the SRR design would produce a significant increase in the relative frequency shift at the expense of a decreased notch depth (see Figure 5a). The DGS could further improve the relative frequency shift and simultaneously increase the notch depth (see Figure 5b). As shown in Figure 5b, the DSRR structure had little influence on the sensor sensitivity but could increase the notch depth, thereby improving the sensor resolution.

## 3. Experimental Validation

**Figure 8:**
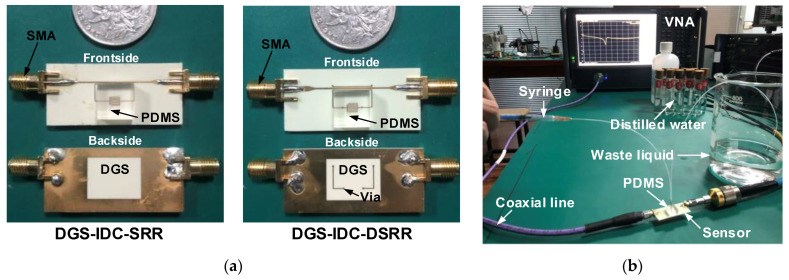
Photographs of (**a**) fabricated sensors and (**b**) experimental setup.

To verify the sensor functionality, the latter two sensor prototypes were fabricated using the PCB technology, as shown in Figure 8a. The PDMS microfluidic channel chip was produced according to a previous design and attached to the sensing area. Two 50 Ω SMA connectors were mounted on the microstrip line. Figure 8b shows a photograph of the experimental setup. The sensor responses were recorded by the Keysight D5234B Vector Network Analyzer (VNA) (Keysight Technology, DE, USA). Ethanol–water solutions with different ethanol volume fractions were prepared to be used as the liquid under test (LUT) and slowly pushed into the PDMS microfluidic channel through a syringe under a pressure lower than 300 mbar to prevent rupture. In order to reduce the operating error, the channel was first emptied and then dried with a hot-air blower before injecting the liquid sample. The stop-flow technique was adopted to avoid bubbles that would affect the accuracy of the experiment as much as possible.

**Figure 9:**
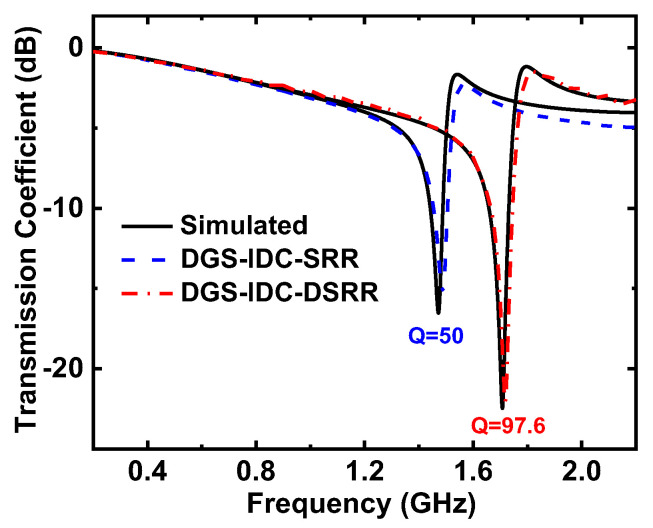
Measured transmission coefficient of the proposed sensor with the PDMS microfluidic channel.

Figure 9 shows the transmission coefficients of the proposed sensor with the PDMS microfluidic channel substrate. The measured results were in good agreement with the simulated results. As the PDMS substrate was attached, both the resonant frequency and the notch depth of the sensor decreased. It is evident that the DGS-IDC-DSRR could provide a larger notch depth than the DGS-IDC-SRR. Here, the quality factor of the sensor was defined as the ratio of the resonant frequency to the −3 dB bandwidth. It was determined that these two sensors had quality factors of 50 and 97.6, respectively. It is evident that the sensor quality factor can be improved by replacing the SRR with a DSRR, thereby improving the sensor resolution.

**Figure 10:**
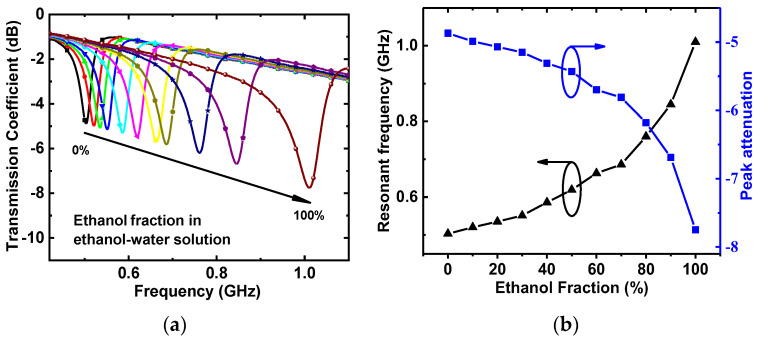
(**a**) Transmission coefficient and (**b**) corresponding resonant frequency and peak attenuation of the DGS-IDC-SRR-based sensor.

Then, ethanol–water solutions with an ethanol volume fraction increasing from 0% to 100% by steps of 10% were injected into the microfluidic channel, and the transmission coefficients were recorded using the VNA. Figure 10a shows the measured responses of the proposed DGS-IDC-SRR-based sensor. As the ethanol fraction increased, the liquid permittivity decreased, and the resonant frequency increased from 0.503 GHz to 1.01 GHz. At the same time, the notch depth decreased from −4.9 dB to −7.78 dB with the increasing ethanol fraction, as shown in Figure 10b.

The relationship between the liquid permittivity $\varepsilon =\varepsilon^{\prime }-j\varepsilon^{\prime \prime }$ and the sensor responses including the resonant frequency $f_{0}$ and the peak attenuation $\left|S_{21}\right|$ is plotted in Figure 10b. A simple mathematical model was established as

(3) $$\left[\begin{array}{c} \Delta f_{0}\\ \Delta \left|S_{21}\right| \end{array}\right]=\left[\begin{array}{cc} m_{11} & m_{12}\\ m_{21} & m_{22} \end{array}\right]\left[\begin{array}{c} \Delta \varepsilon^{\prime }\\ \Delta \varepsilon^{\prime \prime } \end{array}\right]+\left[\begin{array}{cc} n_{11} & n_{12}\\ n_{21} & n_{22} \end{array}\right]\left[\begin{array}{c} \Delta {\varepsilon^{\prime }}^{2}\\ \Delta {\varepsilon^{\prime \prime }}^{2} \end{array}\right]$$

where $\Delta \varepsilon^{\prime }=\varepsilon_{\mathrm{sam}}^{\prime }-\varepsilon_{\mathrm{ref}}^{\prime }$, $\Delta \varepsilon^{\prime \prime }=\varepsilon_{\mathrm{sam}}^{\prime \prime }-\varepsilon_{\mathrm{ref}}^{\prime \prime }$, $\Delta f_{0}=f_{\mathrm{sam}}-f_{\mathrm{ref}}$, and $\Delta \left|S_{21}\right|={\left|S_{21}\right|}_{\mathrm{sam}}-{\left|S_{21}\right|}_{\mathrm{ref}}$. The subscripts of “sam” and “ref” represent the cases when the liquid sample was loaded and when the channel was filled with pure ethanol. The complex permittivity of ethanol aqueous solutions with different ethanol fractions reported in [28] were used as the reference values. The element values of the matrices in equation (3) were obtained using the nonlinear least square curve fitting tool in Matlab software (MathWorks, Natick, MA, USA) [16]. By substituting the obtained parameters into (3), the complete mathematical relationship can be written as

(4) $$\left[\begin{array}{c} \Delta f_{0}\\ \Delta \left|S_{21}\right| \end{array}\right]=\left[\begin{array}{cc} -0.01376 & -0.01271\\ 0.08104 & 0.08652 \end{array}\right]\left[\begin{array}{c} \Delta \varepsilon^{\prime }\\ \Delta \varepsilon^{\prime \prime } \end{array}\right]+\left[\begin{array}{cc} 9.154\times 10^{-5} & -0.003231\\ -5.891\times 10^{-4} & 0.02581 \end{array}\right]\left[\begin{array}{c} \Delta {\varepsilon^{\prime }}^{2}\\ \Delta {\varepsilon^{\prime \prime }}^{2} \end{array}\right]$$

By inverse operation, the complex permittivity of a liquid can be obtained by

(5) $$\left[\begin{array}{c} \Delta \varepsilon_{sam}^{\prime }\\ \Delta \varepsilon_{sam}^{\prime \prime } \end{array}\right]=\left[\begin{array}{cc} 597.037 & 96.073\\ -319.876 & -45.148 \end{array}\right]\left[\begin{array}{c} \Delta f_{0}\\ \Delta \left|S_{21}\right| \end{array}\right]+\left[\begin{array}{cc} 950.967 & -18.486\\ -289.210 & 4.6080 \end{array}\right]\left[\begin{array}{c} \Delta f_{0}{}^{2}\\ \Delta {\left|S_{21}\right|}^{2} \end{array}\right]$$

**Figure 11:**
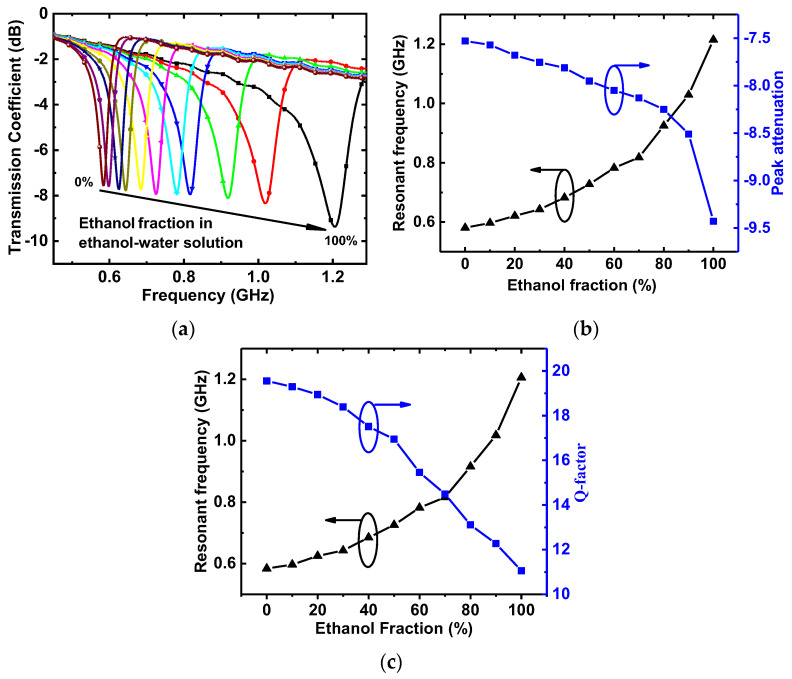
(**a**) Transmission coefficient and (**b**) corresponding resonant frequency and peak attenuation (**c**) resonant frequency and Q-factor of the DGS-IDC-DSRR-based sensor.

Similarly, the transmission coefficients of the proposed DGS-IDC-DSRR-based sensor were measured, as shown in Figure 11. As the ethanol fraction of the ethanol–water solution increased, the resonant frequency increased from 0.584 GHz to 1.206 GHz, while the notch depth decreased from −7.6 dB to −9.4 dB. By following the similar procedure described above, the mathematical relationship between the liquid permittivity and the sensor responses was established as

(6) $$\left[\begin{array}{c} \Delta \varepsilon_{sam}^{\prime }\\ \Delta \varepsilon_{sam}^{\prime \prime } \end{array}\right]=\left[\begin{array}{cc} 117.067 & 21.796\\ -75.155 & -5.678 \end{array}\right]\left[\begin{array}{c} \Delta f_{0}\\ \Delta \left|S_{21}\right| \end{array}\right]+\left[\begin{array}{cc} 262.504 & -0.7912\\ -73.204 & -3.0222 \end{array}\right]\left[\begin{array}{c} \Delta f_{0}{}^{2}\\ \Delta {\left|S_{21}\right|}^{2} \end{array}\right]$$

**Figure 12:**
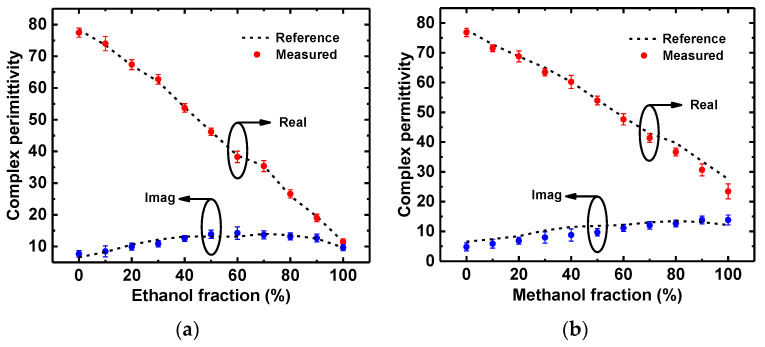
Retrieved complex permittivity of (**a**) an ethanol-water solution and (**b**) a methanol–water solution.

Then, the measured resonant frequency and peak attenuation in the presence of different ethanol fractions were substituted into (6) to retrieve the complex permittivity of the liquid sample, with the retrieved liquid permittivity shown in Figure 12a. Methanol–water solutions with different volumes of the methanol fractions were also tested, and the corresponding measured resonant frequency and peak attenuation were input in (6) to retrieve their complex permittivity. The retrieved liquid permittivity is plotted in Figure 12b, and it is shown that the retrieved values were reasonably consistent with the reference values. Note that the measurements were conducted for three times to demonstrate their repeatability with this sensor repeatability.

**Figure 13:**
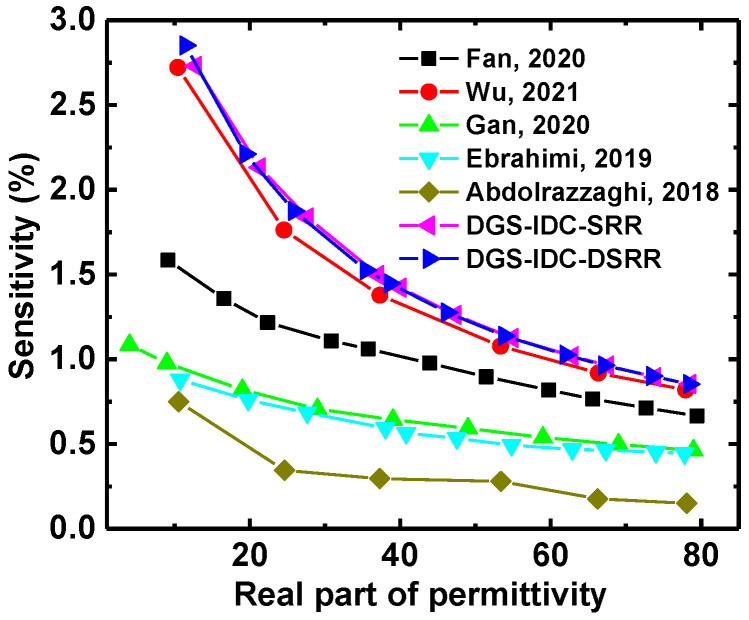
Comparison between the sensitivity of the proposed sensors and that of state-of-the-art devices based on measured data [5,6,8,10,29].

Finally, the proposed sensors were compared with previous designs. Here, the sensor sensitivity is defined as

(7) $$S_{\varepsilon_{r}^{\prime }}=\frac{\Delta f}{f_{\mathrm{unloaded}}\cdot \left(\varepsilon_{r}^{\prime }-1\right)}=\frac{f_{\mathrm{unloaded}}-f_{\varepsilon_{r}^{\prime }}}{f_{\mathrm{unloaded}}\cdot \left(\varepsilon_{r}^{\prime }-1\right)}$$

where $f_{\mathrm{unloaded}}$ and $f_{\varepsilon_{r}^{\prime }}$ represent the resonant frequencies of the sensors unloaded and loaded, respectively. As shown in Figure 13, the proposed sensors exhibited a performance comparable to that of state-of-the-art devices. The performance indicators including liquid sample volume (S.V.), relative electrical size and average sensitivity $S_{\mathrm{avg}}$ of the proposed sensors and previous designs are summarized in Table 1. The average sensitivity of the sensor denotes the average value of the sensitivities shown in Figure 13. It is evident that the proposed sensors exhibited significant advantages in sensitivity in comparison with previous designs. Although the sensor developed in [6] showed a comparable sensitivity, the high required amount of liquid would limits its application range (e.g., glucose level sensing).

## 4. Conclusions

This paper proved that the introduction of an interdigital structure, a defected ground structure and an improved SRR structure can improve the sensitivity and notch depth of traditional SRR-based sensors. It was deduced from the results that the two latter structures influenced the line impedance, increasing the amplitude of the S-parameter. Moreover, the introduced structure only uses the metal ground under the substrate and does not change the plane area of the sensors. According to the electric field distribution, a microfluidic channel covering the meander gap of the SRR was designed. Two prototypes of the proposed sensor were fabricated and tested, and the expected results were obtained. The research results provide a useful reference for the design of three-dimensional resonant sensors with the help of metal vias. At the same time, the possibility of increasing the peak attenuation of the sensor through a structural improvement was proved without introducing an additional plane area or an external active circuit.

## Figures and Tables

**Table 1:** Comparison of the proposed sensor with recent designs.

Ref.	Resonant Type	$f_{o}\;\left(GHz\right)$	S.V. (μL)	$\mathrm{Relative}\;\mathrm{Size}\;(\lambda_{g}^{2})$	$S_{avg}\;(\%)$
[8]	CSRR	2.226	0.52	0.249 × 0.435	0.98
[6]	CSRR	2.45	1.67	0.536 × 0.334	1.444
[7]	EIT-like	0.97	1000	0.13 × 0.13	0.05
[10]	MCSRR	1.62	0.39	0.763 × 0.490	0.626
[5]	Series LC	1.91	0.39	N/A	0.635
[29]	SRR	2.5	5	1.167 × 0.375	0.27
This work	DGS-IDC-SRR	1.49	0.68	0.326 × 0.166	1.430
	DGS-IDC-DSRR	1.72	0.68	0.373 × 0.173	1.461
